# Purification of functional baculovirus particles from silkworm larval hemolymph and their use as nanoparticles for the detection of human prorenin receptor (PRR) binding

**DOI:** 10.1186/1472-6750-11-60

**Published:** 2011-06-02

**Authors:** Tatsuya Kato, Fumiaki Suzuki, Enoch Y Park

**Affiliations:** 1 Laboratory of Biotechnology, Faculty of Agriculture, Shizuoka University, 836 Ohya, Suruga-ku, Shizuoka 422-8529, Japan; 2 Laboratory of Animal Biochemistry, Faculty of Applied Biological Sciences, Gifu University, 1-1 Yanagido, Gifu 501-1193, Japan; 3 Integrated Bioscience Section, Graduate School of Science and Technology, Shizuoka University, 836 Ohya, Suruga-ku, Shizuoka 422-8529, Japan

**Keywords:** BmNPV, human prorenin receptor, silkworm, display, ELISA

## Abstract

**Background:**

Baculovirus, which has a width of 40 nm and a length of 250-300 nm, can display functional peptides, receptors and antigens on its surface by their fusion with a baculovirus envelop protein, GP64. In addition, some transmembrane proteins can be displayed without GP64 fusion, using the native transmembrane domains of the baculovirus. We used this functionality to display human prorenin receptor fused with GFP_uv _(GFP_uv_-hPRR) on the surface of silkworm *Bombyx mori *nucleopolyhedrovirus (BmNPV) and then tested whether these baculovirus particles could be used to detect protein-protein interactions.

**Results:**

BmNPV displaying GFP_uv_-hPRR (BmNPV-GFP_uv_-hPRR) was purified from hemolymph by using Sephacryl S-1000 column chromatography in the presence of 0.01% Triton X-100. Its recovery was 86% and the final baculovirus particles number was 4.98 × 10^8 ^pfu. Based on the results of enzyme-linked immunosorbent assay (ELISA), 3.1% of the total proteins in BmNPV-GFP_uv_-hPRR were GFP_uv_-hPRR. This value was similar to that calculated from the result of western blot by a densitometry (2.7%). To determine whether BmNPV-GFP_uv_-hPRR particles were bound to human prorenin, ELISA results were compared with those from ELISAs using protease negative BmNPV displaying β1,3-*N*-acetylglucosaminyltransferase 2 fused with the gene encoding GFP_uv _(GGT2) (BmNPV-*CP*^-^-GGT2) particles, which do not display hPRR on their surfaces.

**Conclusion:**

The display of on the surface of the BmNPV particles will be useful for the detection of protein-protein interactions and the screening of inhibitors and drugs in their roles as nanobioparticles.

## Background

Baculovirus has been used widely to express recombinant proteins in insect cells and larvae [1,2]. *Autographa californica *multiple nucleopolyhedrovirus (AcMNPV) has been the most commonly used baculovirus for recombinant protein production [3]. Baculovirus infection can be divided to three distinct phases, early, late and very late phase. While budding virus (BV) is produced in the late phase, the occlusion derived virus (ODV) form is produced in the very late phase. The BV form has a width of 40 nm and a length of 250-300 nm [4] has been used as a nanoparticle [5,6] in a baculovirus surface displaying system. ODV form can also be used as nanoparticles, because ODV-polyhedrin particles are resistant to heat and light inactivation, whereas BV is more sensitive to environment. Cultured insect cells are used to produce and amplify recombinant AcMNPV, which means that large-scale cultivation of insect cells is also needed to produce recombinant baculovirus particles. By contrast, silkworm *Bombyx mori *nucleopolyhedrovirus (BmNPV) was recently used for the large-scale production of recombinant proteins and baculovirus particles owing to its ability to infect silkworm larvae and pupae [2]. Silkworms can rapidly produce a high level of recombinant proteins and baculovirus particles with BmNPV bacmids [7,8]. For example, ~2.2 mg of purified human α2,6-sialyltransferase was obtained from only 11 silkworm larvae injected with recombinant bacmid harboring α2,6-sialyltransferase gene [9]. Predicted amino acid sequences of corresponding ORFs of BmNPV are closely related to those of AcMNPV (~90% relatedness) [10].

Once established, the baculovirus display system can be used to produce baculoviruses displaying functional peptides, receptors and antigens that enable the delivery of heterologous gene expression in mammalian cells and tissues, as well as the production of antibodies and vaccines [11-13]. Baculovirus has an envelope protein (GP64) that comprises an N-terminal signal peptide, a mature domain, a transmembrane domain and a short cytoplasmic domain at its C-terminus. Heterogeneous peptides and proteins can be displayed on the surface of baculovirus envelope by fusion with either the full-length GP64 or its transmembrane and cytoplasmic domains [14]. Some transmembrane proteins can be also displayed on the surface of baculovirus without fusing with any domain [15,16]. Several enzymes and receptors can be displayed with its native form [17] and receptor-displaying baculoviruses are used for specific protein-protein interaction detection and the expression cloning of CD2 cDNA from cDNA expression libraries by magnetic separation. As an alternative baculovirus display system, baculovirus capsid display has been also established by fusion with a nucleocapsid protein, VP39, for transduction imaging [18]. The baculovirus display system is also becoming more important in the life sciences as an addition to the baculovirus expression system.

Recently, many studies have reported that human prorenin receptor (hPRR), and its mechanism of binding to renin/prorenin, involves the generation or action of angiotensin, leading to numerous cardiovascular diseases [19-21]. Consequently, the development of hPRR receptor blockers is currently receiving considerable attention. Also, an understanding of the functional properties of hPRR through detailed biochemical and biophysical analysis is required. In a previous study, hPRR fused with GFP_uv _at its N-terminus (GFP_uv_-hPRR) was expressed and purified from the fat body of silkworm larvae infected with recombinant baculovirus [22,23]. However, the binding capacity of purified GFP_uv_-hPRR to human prorenin was reduced compared with that before purification. Possible reasons might be that the protein structure is broken or that the recognition sites become buried inside the molecule during purification. In the current study, we attempted to display GFP_uv_-hPRR on the surface of BmNPV and to purify the baculovirus particles from silkworm larval hemolymph using size-exclusion chromatography (SEC). Purified baculovirus particles were characterized and used to detect the interaction of GFP_uv_-hPRR and human prorenin by an enzyme-linked immunosorbent assay (ELISA).

## Results

### Improvement of purification efficiency of BmNPV particles

**Figure 1:**
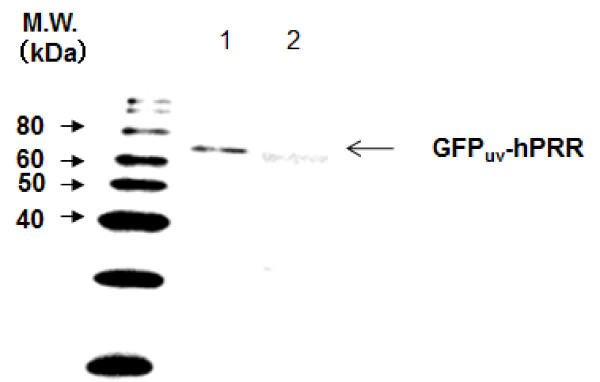
**GFP_uv_-hPRR in purified BmNPV-GFP_uv_-hPRR particles**. Proteins were separated by a 12% SDS-PAGE gel and GFP_uv_-hPRR was detected by Western blot. Lane 1: purified BmNPV-GFP_uv_-hPRR particles; lane 2: purified BmNPV-*CP*^-^-GGT2 particles.

As detailed in a previous report [8], hPRR-displayed BmNPV (BmNPV-hPRR) was produced in silkworm larvae and purified by SEC. Its SEC recovery efficiency was ~35% and decreased by 4% with ultracentrifugation to concentrate the baculovirus particles. Final BmNPV particle concentration was 1.6 × 10^8 ^pfu ml^-1 ^[8]. In the current study, BmNPV-GFP_uv_-hPRR was produced and purified using the same methods as detailed in [8]. GFP_uv_-hPRR was detected in purified BmNPV-GFP_uv_-hPRR particles, but not in purified protease negative BmNPV-*CP*^-^-GGT2 particles, which harbor the gene encoding human β1,3-*N*-acetylglucosaminyltransferase 2 fused with the gene encoding GFP_uv _(GGT2) instead of the gene encoding GFP_uv_-hPRR (Figure 1) [24]. The faint band (~65 kDa) found on analysis of the purified BmNPV-*CP*^-^-GGT2 particles is likely to represent an, as yet, unspecified molecule. This kind of band could sometimes be detected using anti-FLAG. The estimated molecular weight of the GFP_uv_-GGT2 fusion protein is ~77 kDa, larger than that of GFP_uv_-hPRR (69 kDa), indicating this band is not derived from GGT2. Recovery of BmNPV-GFP_uv_-hPRR particles was 29% using SEC.

To improve the recovery efficiency of BmNPV-GFP_uv_-hPRR, BmNPV-GFP_uv_-hPRR particles were suspended with PBS containing 0.01% each of the detergents Triton X-100, Tween 20 and PF-68, after ultracentrifugation. Using purification, the recovery efficiency of baculovirus particles was 0.9% without detergent. Using PBS containing 0.01% of each detergent as a suspension buffer after ultracentrifugation, particle purity and the recovery efficiency improved. For example, when PF-68 was used purity was increased higher 1000 times to that of hemolymph, but recovery efficiency was low (1.4%). Using Triton X-100 the recovery ratio was approximately ten times more than for PBS alone and particle purity was 475 times more than that of hemolymph (Table 1).

**Table 1:** Purification of BmNPV-GFP_uv_-hPRR particles by Sephacryl S-1000 column chromatography

	Volume (ml)	**Titer (pfu ml**^**-1**^**)**	Virus (pfu)	**Protein (mg ml**^**-1**^**)**	**Purity (pfu mg**^**-1 **^**protein)**	Recovery (%)
Hemolymph	7	2.38 × 10^8^	1.67 × 10^9^	37.3	6.38 × 10^6 ^(1)	100
Sephacryl S-1000 chromatography	30	1.67 × 10^7^	4.86 × 10^8^	0.011	1.52 × 10^9 ^(238)	29
**After ultracentrifugation**						
Suspended with PBS	0.5	2.97 × 10^7^	1.49 × 10^7^	0.111	2.68 × 10^8 ^(42)	0.9
Suspended with 0.01% Triton X-100	0.5	3.33 × 10^8^	1.66 × 10^8^	0.110	3.03 × 10^9 ^(475)	10
Suspended with 0.01% Tween 20	0.5	1.51 × 10^8^	7.55 × 10^7^	0.071	2.13 × 10^9 ^(334)	4.5
Suspended with 0.01% PF-68	0.5	4.60 × 10^8^	2.30 × 10^7^	0.059	7.80 × 10^9 ^(1,222)	1.4

Sephacryl S-1000 chromatography was then performed using PBS containing each concentration (0.01 or 0.1%) of Triton X-100 or Tween 20. The recovery efficiency of BmNPV-GFP_uv_-hPRR particles was improved in the presence of detergent. For example, in the presence of 0.01% Triton X-100, its efficiency was 86%, which was threefold more than using PBS alone (Table 2).

**Table 2:** Purification of BmNPV-GFP_uv_-hPRR particles by Sephacryl S-1000 column chromatography with detergent-containing PBS

	Volume (ml)	**Titer (pfu ml**^**-1**^**)**	Virus (pfu)	Recovery (%)
Hemolymph	7	2.38 × 10^8^	1.67 × 10^9^	100
Sephacryl S-1000 (PBS)	30	1.67 × 10^7^	4.86 × 10^8^	29
Hemolymph	7	8.30 × 10^7^	5.81× 10^8^	100
Sephacryl S-1000 (0.01% Triton X-100 in PBS)	30	1.66 × 10^7^	4.98 × 10^8^	86*
Hemolymph	7	3.33 × 10^8^	2.33 × 10^8^	100
Sephacryl S-1000 (0.1% Triton X-100 in PBS)	30	3.04 × 10^6^	9.12 × 10^7^	41
Hemolymph	7	2.38 × 10^8^	1.67 × 10^9^	100
Sephacryl S-1000 (0.01% Tween 20 in PBS)	30	2.29 × 10^7^	6.87 × 10^8^	41

* This purification protocol was repeated several times and typical result is shown.

**Figure 2:**
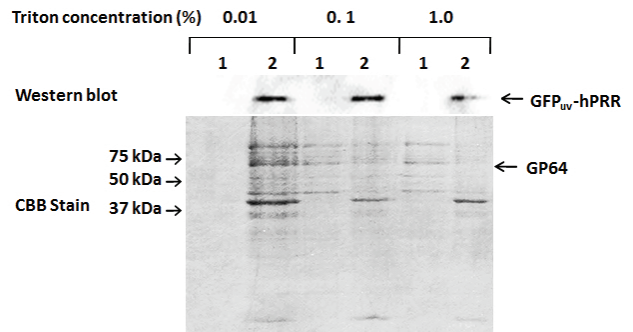
**Treatment of purified BmNPV-GFP_uv_-hPRR particles with each concentration of Triton X-100**. Purified BmNPV-GFP_uv_-hPRR particles were suspended with 0, 0.01, 0.1 or 1% of TritonX-100, respectively and supernatant (1) and pellet (2) were separated by ultracentrifugation. Proteins were separated by a 12% SDS-PAGE gel and GFP_uv_-hPRR was detected by Western blot and CBB stain.

However, its recovery efficiency decreased in the presence of 0.1% Triton X-100, which indicates that a high detergent concentration was deleterious to BmNPV particles. To confirm this effect, purified BmNPV particles were treated with each concentration (0.01, 0.1 and 1%) of Triton X-100. In the presence of 1% Triton X-100, GFP_uv_-hPRR amount in the pellet fraction was lower than that of 0.01 or 0.1% treatment (Figure 2). Proteins were solubilized less under the 0.01% TritonX-100 treatment compared with the 0.1% or 1% Triton X-100 treatment. However, as the concentration of Triton X-100 was increased, more amounts of GP64 and other proteins were solubilized into the supernatant fraction. It was previously reported that GFP_uv_-hPRR was not solubilized from the microsome fraction of silkworm fat body efficiently by Triton X-100 [23]. Considering the result of Table 2 0.01% TritonX-100 treatment are milder to baculovirus envelope than 0.1% or 1% Triton X-100 treatment.

### Confirmation of surface display of GFP_uv_-hPRR on the BmNPV particles with proteinase K

**Figure 3:**
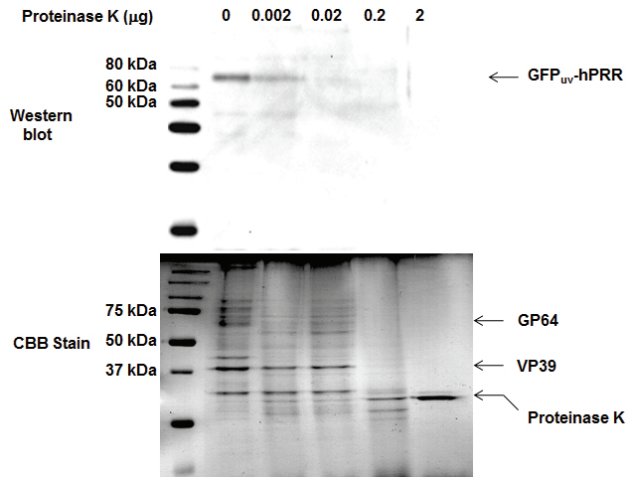
**Proteinase K treatment of purified BmNPV-GFP_uv_-hPRR particles**. Purified BmNPV-GFP_uv_-hPRR particles were suspended with either 0, 0.002, 0.02, 0.2 or 2 μg of proteinase K, respectively, and incubated at room temperature for 15 min. Proteins were separated by a 12% SDS-PAGE gel and GFP_uv_-hPRR was detected by Western blot and CBB stain.

To confirm the surface display of GFP_uv_-hPRR on the BmNPV particles, purified BmNPV-GFP_uv_-hPRR particles were treated with proteinase K (0.002, 0.02, 0.2 and 2 μg). If GFP_uv_-hPRR was displayed on the surface of the BmNPV particles, it would be degraded by a less amount of proteinase K than would the VP39 protein, which is a nucleocapsid protein localized inside BmNPV particles. In the presence of 0.02 μg proteinase K, VP39 was detected, but hPRR was not (Figure 3), suggesting that displayed GFP_uv_-hPRR on the surface of BmNPV particles had been degraded. In the presence of 0.2 or 2 μg of proteinase K, hPRR and VP39 were both degraded.

### Quantification of GFP_uv_-hPRR displayed on the surface of BmNPV particles

**Figure 4:**
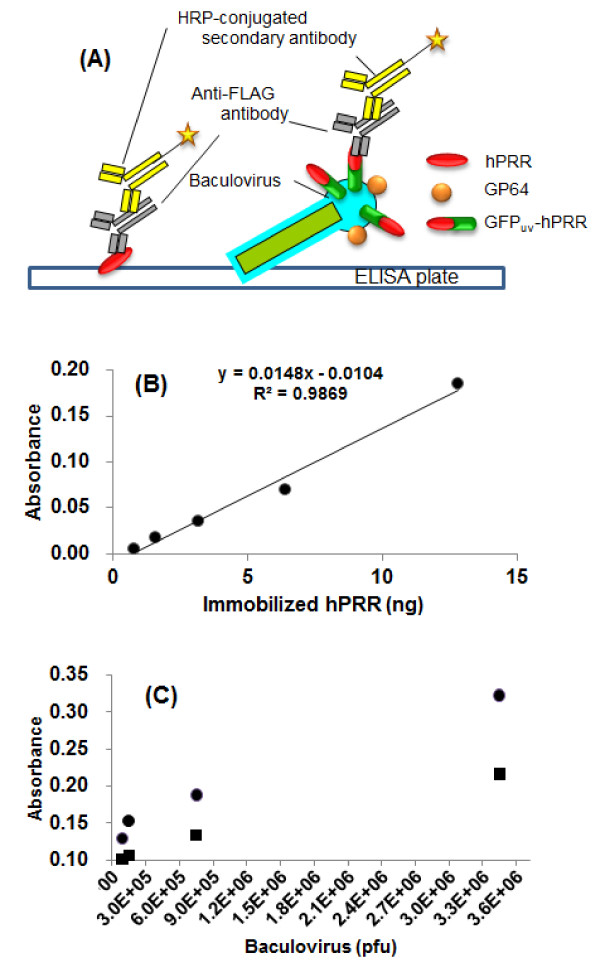
**Detection of hPRR by ELISA**. (A) Illustration of Detection of hPRR by ELISA. For determination of immobilized hPRR (left), purified hPRR were immobilized on a plate. And then, mouse anti-FLAG M2 antibody was incubated for binding hPRR and anti-FLAG M2 antibody. HRP-conjugated anti-mouse IgG antibody was used for detection of binding hPRR and anti-FLAG M2 antibody. Based on this hPRR calibration curve, the amount of displayed GFP_uv_-hPRR was determined (right). BmNPV-GFP_uv_-hPRR particles were immobilized on a plate. And then, mouse anti-FLAG M2 antibody was incubated for binding hPRR and anti-FLAG M2 antibody. Detection of hPRR was carried out by HRP-conjugated anti-mouse IgG antibody. (B) Calibration curve of purified hPRR by ELISA. (C) Detection of hPRR on the surface of BmNPV-GFP_uv_-hPRR particles. Closed circles and closed squares denote purified BmNPV GFP_uv_-hPRR particles and purified BmNPV-*CP*^-^-GGT2 particles, respectively.

To quantify GFP_uv_-hPRR displayed on the surface of BmNPV particles, an ELISA technique was used. BmNPV-GFP_uv_-hPRR particles were immobilized in the wells and hPRR was detected by ELISA (Figure 4A). BmNPV-*CP*^-^-GGT2, which does not display hPRR on the surface of BmNPV particles, was used as a negative control. First, hPRR purified from insect cells [25] was used as a standard and a calibration curve of hPRR was made. A liner relationship was observed between 0 and 13 ng of hPRR with a correlation coefficient of 0.9869 (Figure 4B). Absorbance was higher for BmNPV-GFP_uv_-hPRR than for BmNPV-*CP*^-^-GGT2, indicating that BmNPV-GFP_uv_-hPRR was displayed on the surface of BmNPV particles (Figure 4C). Based on this result, 8.1 ng of GFP_uv_-hPRR was displayed on the surface of 260 ng of BmNPV-GFP_uv_-hPRR proteins and ~3.1% of total proteins in BmNPV-GFP_uv_-hPRR was GFP_uv_-hPRR. This value was similar to that calculated by densitometry from the results of a Western blot (Figure 1; 2.7%).

### Application of GFP_uv_-hPRR displaying BmNPV to protein-protein interaction detection

**Figure 5:**
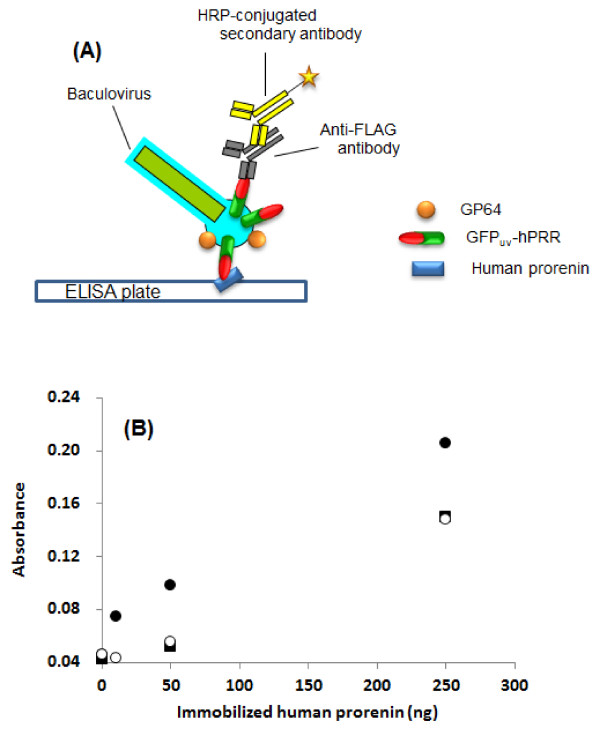
**Detection of binding of GFP_uv_-hPRR to human prorenin by ELISA**. (A) Illustration of detection of binding of GFP_uv_-hPRR to human prorenin by ELISA. In prorenin-hPRR binding detection experiment, human prorenin was immobilized on a plate. Approximately 3.5 × 10^6 ^pfu of BmNPV particles were added into the plate and each well and this plate was incubated. Detection of BmNPV binding to human prorenin was the same method as hPRR detection as shown in Figure 4. (B) Detection of binding of BmNPV-GFP_uv_-hPRR particles to human prorenin. Human prorenin was immobilized in a 96-well plate and incubated together with BmNPV-GFP_uv_-hPRR particles. BmNPV-GFP_uv_-hPRR particles binding to human prorenin were detected by ELISA. Closed circles, closed squares and open circles denote purified BmNPV GFP_uv_-hPRR particles, purified BmNPV-*CP*^-^-GGT2 particles and PBS (pH 6.2), respectively.

Previously, Sakihama *et al*. reported that specific interactions between CD2-displaying AcMNPV particles and CD58-displaying AcMNPV particles could be detected by ELISA and that baculovirus surface display technology was therefore applicable to protein-protein interaction detection [17]. The binding of hPRR to its ligand, human prorenin, was detected using BmNPV-GFP_uv_-hPRR particles by ELISA. Human prorenin was immobilized on the wells and then incubated with purified BmNPV-GFP_uv_-hPRR particles (Figure 5A). GFP_uv_-hPRR displaying on the surface of BmNPV particles bind to human prorenin immobilized onto the plate, and then are detected by ELISA using mouse anti-FLAG M2 and HRP-conjugated anti-mouse IgG as primary and secondary antibodies (Figure 5B). Significant absorbance of BmNPV-GFP_uv_-hPRR was detected compared to only a buffer or BmNPV-*CP*^-^-GGT2, which does not display hPRR on its surface. This result indicates that BmNPV surface display is applicable to protein-protein interaction detection; in addition ELISAs based on this BmNPV system could be used in the screening of hPRR blockers.

## Discussion

Baculovirus has been used widely for recombinant protein production, and recently as a surface display system. Some transmembrane proteins are displayed on the baculovirus surface without any fusion with the baculovirus envelope protein [15,16,26]. Baculoviruses that display transmembrane proteins on their surfaces are suitable for use in ELISAs, for the expression cloning of a gene from a cDNA library and for making proteoliposomes [17,27]. In particular, baculovirus particles can be used as probes for the expression cloning of receptors and ligands from cDNA libraries using magnetic sorting and flowcytometric analysis [17]. This method enabled the cloning of CD2 cDNA from a human T-cell cDNA library using CD58 displaying AcMNPV particles.

Previously, we reported the production of high titers of BmNPV displaying hPRR in silkworm larvae [8]. When the BmNPV-GFP_uv_-hPRR particles were purified by Sephacryl S-1000 column chromatography without using detergent, the recovery yield of particles was only 30% [8], which was similar to that of this work (Tables 1 and 2). In the current study, we were able to purify hPRR-displaying BmNPV particles from the hemolymph of silkworm larvae with high recovery in the presence of 0.01% Triton X-100 (Table 2). In the case of virus-like particles (VLP), these can be stabilized and prevented from aggregation and precipitation by using non-ionic detergents and other compounds [28-30]. Our results suggest that the high level of recovery of baculovirus particles in the presence of Triton X-100 might result from inhibition of particle aggregation by Triton X-100. Moreover, non-ionic detergents can prevent the adsorption of protein to surfaces [31,32]. In our experiments, the optimal use of a non-ionic detergent contributed the high recovery of baculovirus particles. However, when a higher-than-optimal amount of detergent was used (>0.01%), the displayed protein was solubilized (Figure 2). These results suggest that the deleterious effect of high detergent concentration during purification of BmNPV-GFP_uv_-hPRR particles was caused by the loss of envelope proteins on the surface of the particles. In Table 1, although protein concentration of Triton X-100-suspended fraction was the same as that of PBS-suspended one, purity of Triton X-100-suspended BmNPV particles and recovery ratio were 11% higher than that of PBS-suspended ones. This suggests that the presence of 0.01% Triton X-100 is effective for the purification of BmNPV particles from silkworm larval hemolymph.

Aggregation is a major problem in the production, purification and concentration of viruses [33,34]. It has also been reported that virus instability is due to the aggregation of virus particles, which results in their precipitation [35,36]. To obtain high titer of virus stocks, viruses have to be concentrated. However, such high titers are prone to aggregate during purification and concentration steps. In the current study, we were able to purify high titers of BmNPV particles displaying hPRR from the hemolymph of silkworm larvae without aggregation in the presence of 0.01% Triton X-100 and to omit the concentration step. Silkworms can produce higher titers of baculoviruses than cultured cells, Sf-9 and Sf-21 cells. The method of using silkworm larvae and SEC in the presence of Triton X-100 is therefore useful for the high-titer preparation of functional BmNPV particles displaying heterogeneous transmembrane proteins or ligands.

In this study, ~3% of total proteins of BmNPV particles were displayed as GFP_uv_-hPRR. GP64 is a major protein in the envelope of baculoviruses. The display of heterogeneous proteins and peptides can be also achieved by the fusion with GP64 [14,37-39]. More hPRR can be displayed by the fusion with the transmembrane and cytoplasmic domains of GP64 than with those of hPRR because of the abundance of GP64 in the baculovirus envelope. The envelope glycoprotein of human immunodeficiency virus (HIV) can be incorporated into HIV virus-like particles by the replacement of its native transmembrane and cytoplasmic domains with those of GP64 [39]. The cytoplasmic domain might be important for incorporating the HIV envelope protein into the lipid bilayer of envelope viruses and virus-like particles and for it to stably reside in its envelope. The cytoplasmic domain of the transmembrane domain might be also important for displaying on the surface of baculovirus particles.

Displaying GFP_uv_-hPRR on its surface was detected by ELISA using mouse anti-FLAG M2 and HRP-conjugated anti-mouse IgG antibodies without solubilization and purification of hPRR. GFP_uv_-hPRR on the surface of BmNPV particles was confirmed as an active form by binding to human prorenin immobilized onto the plate. However, accuracy of ELISA was not high enough, because of small difference of detected absorbance between BmNPV-GFP_uv_-hPRR and negative control (BmNPV-*CP*^-^-GGT2). This ELISA can be improved by using anti-GP64 antibody as a primary antibody because GP64 is displayed on the surface of BmNPV particles far more than GFP_uv_-hPRR. Moreover, this ELISA has to be improved for more precise binding analysis. For example, the activation of human prorenin by its binding to hPRR might be detected by immobilization of the hPRR-displaying BmNPV particles onto the plate. This ELISA is simple and convenient compared to previous method [40] and a new ELISA for the analysis of hPRR and hPRR blockers screening can be established based on BmNPV surface display system.

## Conclusion

We have shown here that the BmNPV surface display system can be utilized in protein-protein interaction detection. A large-scale production of BmNPV particles can be performed using silkworm larvae and high titer of BmNPV particles can be purified from hemolymph using SEC with a high recovery ratio. This BmNPV-silkworm system could be used for the efficient and effective application of baculovirus particles as nanoparticles because of the scale on which baculovirus particles can be produced and the low cost and simple protocol of that production.

## Methods

### Production and purification of recombinant BmNPV in silkworm

A recombinant BmNPV, BmNPV-GFP_uv_-hPRR, was constructed by using *E. coli *BmDH10bac [7] according to a previous report [19]. A FLAG sequence was inserted at the N-terminus of hPRR for detection with Western blot and ELISA. A bombyxin signal sequence was connected at the N-terminus of GFP_uv _for GFP_uv_-hPRR to enter into the secretory pathway in the cells of the host. BmNPV-*CP*^-^-GGT2 was constructed according to the protocol described in [24]. Fifth-instar silkworm larvae were injected with 50 μl of a DMRIE-C (Invitrogen)-bacmid mixture. This mixture contained ~5 μg of DNA (bacmid and helper plasmid). To obtain 10 ml of hemolymph, 15 silkworm larvae were reared using an artificial diet, Silkmate 2S (Nihon Nosan, Yokohama, Japan). After rearing for 6-7 days in a 25°C-rearing chamber, larval hemolymph was collected by cutting the caudal leg of the larva in a tube containing 5 μl of 200 mM 1-phenyl-2-thiourea to prevent melanization, and centrifuged at 9000 rpm for 10 min; the hemolymph was kept at -80°C before use. For large-scale production of BmNPV-GFP_uv_-hPRR, 100-fold diluted hemolymph was injected into 15 silkworm larvae.

Purification of BmNPV-GFP_uv_-hPRR was performed according to a previous report [8]. Recovered hemolymph was centrifuged at 8000 × g and supernatant was collected. The supernatant was then loaded onto a Sephacryl S-1000 column (2.6 × 52 cm, GE Healthcare UK Ltd., Buckinghamshire, HP7 9NA, UK) equilibrated with phosphate-buffered saline (PBS, pH 6.2) with each concentration of Triton X-100. Elution was performed at 4°C and monitored by absorbance at 280 and 254 nm. Every 5 ml of the fraction was collected. To concentrate purified BmNPV, fractions containing BmNPV particles were pooled and concentrated by ultracentrifugation at 114000 × g with a 25% sucrose cushion (25% sucrose in 5 mM NaCl and 10 mM EDTA). Particles were suspended by a small volume of PBS (pH 6.2) with each detergent.

### Quantitative real-time PCR to determine the number of baculovirus particles

Quantitative real-time PCR (Q-PCR) was performed according to a previous report [8]. In brief, baculoviral DNA was extracted by using a High Pure Viral Nucleic Acid Kit (Roche Diagnostics K. K., Tokyo, Japan) according to the manufacturer's protocol. The titration assay using Q-PCR was performed by Mx3000P system (Stratagene, La Jolla, CA, USA) and analyzed by MxPro software (Stratagene). The ie-1-specific primers, Bmie-1-F (CCCGTAACGGACCTTGTGCTT) and Bmie-1-R (TTATCGAGATTTATTTACATACAACAAG) were used.

### Treatment of BmNPV particles with Triton X-100 or proteinase K

To treat with Triton X-100, purified BmNPV with PBS (pH 6.2) was concentrated by ultracentrifugation and then suspended with PBS containing each concentration (0.01, 0.1 or 1%) of Triton X-100. Suspensions (8 μg of proteins) were kept at 4°C for 16 hours and ultracentrifuged. The supernatant and pellet were collected separately and sodium dodecyl sulfate-polyacrylamide gel electrophoresis (SDS-PAGE) and Western blot were then performed. To treat with proteinase K, purified BmNPV with PBS (pH 6.2) was concentrated by ultracentrifugation and then suspended with PBS containing 0.01% Triton X-100. Each volume (0.002, 0.02, 0.2 and 2 μg) of proteinase K was added into 3 mg of BmNPV protein and the mixture was incubated at room temperature for 15 min. Proteins of BmNPV particles were analyzed by using SDS-PAGE and Western blot.

### SDS-PAGE and Western blot

Proteins were separated by SDS-PAGE using 12% polyacrylamide gels that were stained with Coomassie Brilliant Blue (CBB) solution. In the case of Western blot, proteins in gels were blotted onto a polyvinylidene fluoride (PVDF) membrane using Mini Trans-Blot Electrophoretic Transfer Cell (Bio-Rad, Hercules, CA, USA). After being blocked in 5% skimmed milk in Tris-buffered saline containing 0.1% Tween 20 (TBST), the membrane was incubated in 1:10000 diluted mouse anti-FLAG M2 antibody (Sigma-Aldrich, St. Louis, MO, USA) for 1 hour. The membrane was washed with TBST, and then incubated in 1:20 000 diluted anti-mouse labeled with horseradish peroxidase (HRP) (GE Healthcare) for 1 hour. Detection was performed using ECL Plus Western blotting reagent (GE Healthcare.). Specific bands were detected using a Fluor-S/MAX multi-imager (Bio-Rad).

### hPRR detection by ELISA

BmNPV particles and purified hPRR were incubated in a 96-well plate at 4°C with 0.1M carbonate buffer (pH 9.6) for 16 hours to become immobilized on the plate. Wells were washed with PBS (pH 7.4) containing 0.01% Tween 20 (PBST) four times. Blocking buffer (PBST containing 3% skimmed milk) was added into each well and plates were incubated at room temperature for 1 hour. Wells were washed with PBS (pH 7.4) containing 0.01% Tween 20 (PBST) four times. Mouse anti-FLAG M2 antibody diluted 1000-fold with Can Get Signal Solution I (TOYOBO, Co. Ltd., Tokyo) was added into each well and the plates were incubated at room temperature with moderately stirring in a 96 well plate shaker (BioShaker V·BR-36, TAITEC, Saitama, Japan) for 1 hour. After wells were washed with PBST four times, HRP-conjugated anti-mouse IgG antibody diluted 5000-fold with PBST was added into each well and the plates were incubated at room temperature with moderately stirring in a 96 well plate shaker (BioShaker) for 1 hour. Wells were washed with PBST four times followed by the HRP reaction. 100 μl of substrate [0.1-mg ml^-1 ^3,3',5,5'-tetramethylbenzidine(TMBZ) in 100-mM sodium acetate, pH 6.0, with 0.2% (v/v) of 30% hydrogen peroxide] was added to each well and left at room temperature for blue-color development. The reaction was stopped by the addition of 50 μl of 1N H_2_SO_4 _solution. The developed color was measured at optical densities (ODs) of 450 nm and 655 nm. The value of OD655 minus OD450 was used as a measure of the amount of hPRR. hPRR that was used as a hPRR standard was purified from insect cells expressing it with anti-FLAG M2 antibody agarose (Sigma-Aldrich Inc., St. Louis, MO, USA) [25]

In the prorenin-hPRR binding detection experiment, human prorenin was incubated in a 96-well plate at 4°C with 50 mM phosphate buffer (pH 7.4) for 16 hours to become immobilized on the plate. Wells were washed with PBS (pH 7.4) containing 0.01% Tween 20 (PBST) four times. Blocking buffer (PBST containing 3% skimmed milk) was added into each well and plates were incubated at room temperature for 1 hour. Wells were washed with PBS T four times. Approximately 3.5 × 10^6 ^pfu of BmNPV particles were added into each well and this plate was incubated at room temperature with moderate stirring in a 96 well plate shaker (BioShaker) for 3 hours. Wells were washed with PBST four times. Detection of BmNPV binding to human prorenin was done using the same method as for hPRR detection.

## Authors' contributions

TK carried out the experimental design, the molecular genetics and biochemical experiments. FS conceived the idea of ELISA application, guided its application on the screening of inhibitors and drugs. EYP directly supervised the project, participated in its experimental design and data interpretation, and was responsible for writing the manuscript. All authors have read and approved the manuscript.
